# Retrospective analysis of antimicrobial resistance and bacterial spectrum of infection in Gabon, Central Africa

**DOI:** 10.1186/1471-2334-13-455

**Published:** 2013-10-02

**Authors:** Abraham S Alabi, Lisa Frielinghaus, Harry Kaba, Katrin Kösters, Michaëla A M Huson, Barbara C Kahl, Georg Peters, Martin P Grobusch, Saadou Issifou, Peter G Kremsner, Frieder Schaumburg

**Affiliations:** 1 Centre de Recherche Médicale de Lambaréné (CERMEL), Lambaréné, Gabon; 2 Institut für Tropenmedizin, Eberhard Karls Universität, Tübingen, Germany; 3 Institute of Medical Microbiology, University Hospital Münster, Münster, Germany; 4 Department of Internal Medicine II, HELIOS-Clinic Krefeld, Krefeld, Germany; 5 Center for Tropical Medicine and Travel Medicine, Department of Infectious, Diseases, Division of Internal Medicine, Academic Medical Center, University of Amsterdam, Amsterdam, The Netherlands

**Keywords:** Antimicrobial resistance, Epidemiology, Africa, Staphylococcus aureus, ESBL

## Abstract

**Background:**

Physicians depend on reliable information on the local epidemiology of infection and antibiotic resistance rates to guide empiric treatment in critically ill patients. As these data are scarce for Central Africa, we performed a retrospective analysis of microbiological findings from a secondary care hospital in Gabon.

**Methods:**

Microbiological reports from 2009 to 2012 were used to assess the non-susceptibility rates of the three most common isolates from six major types of infections (bloodstream, ear-eye-nose-throat, surgical site, skin and soft tissue, urinary tract and wound infection).

**Results:**

A high diversity of pathogens was found, but *Staphylococcus aureus* was predominant in the majority of infections. Overall, the three most prevalent pathogens in children were *S. aureus* (33.7%), *Streptococcus pyogenes* (8.1%) and *Escherichia coli* (4.5%) and in adults *S. aureus* (23.5%), *E. coli* (15.1%) and *Klebsiella pneumoniae* (7.4%). In total, 5.8% (n = 19) of all *S. aureus* isolates were methicillin resistant. The proportion of extended-spectrum beta-lactamase (ESBL) producing Enterobacteriaceae was 15.4% (n = 78), 49.4% of all *K. pneumoniae* were ESBL-producer (n = 42).

**Conclusion:**

The high diversity of potential pathogens and high resistance rates in Gram-negative bacteria challenge a rational empiric use of antibiotics. Countrywide continuous sentinel surveillance is therefore urgently needed.

## Background

The knowledge of the infection epidemiology and the antibiotic resistance patterns is essential to guide optimal empiric treatment in critically ill patients. Antibacterial susceptibility patterns and bacterial spectra vary geographically, highlighting the importance of local surveillance data. In industrialized countries, these data are available at regional, national and international levels; for instance as provided by the European Antimicrobial Resistance Surveillance System (EARSS) database [1]. However, current surveillance data are scarce in Africa [2,3]. The lack of surveillance is alarming as some reports might only show the "tip of the iceberg" such as a 15.5% ciprofloxacin resistance in *Salmonella enterica* Typhi reported from DR Congo [4], 18% penicillin non-susceptibility in *Streptococcus pneumoniae* in remote regions in Gabon [5] and up to 31% colonization rates with extended-spectrum beta-lactamase (ESBL) producing Enterobacteriaceae on hospital admission in Niger [6]. At the moment, these emerging resistances in Africa are a challenge but might become devastating considering the limited access to second line drugs (e. g. vancomycin, carbapenems) in these regions. To provide data for the rational use of antibiotics and tools for target-oriented infection control-measures, we report the antimicrobial resistance rates and bacterial spectra of infections in a secondary care hospital in semi-urban Gabon.

## Methods

### Sample collection

This retrospective analysis of antimicrobial resistance in Gabon, Central Africa consists of data from the microbiology laboratory of the Albert Schweitzer Hospital (HAS) in Lambaréné. All microbiological reports on bacterial pathogens between January 2009 and September 2012 were included. Ethical approval was not obtained as retrospective studies such as our study do not require ethical approval in Gabon. No inclusion or exclusion criteria were applied. The decision to take samples for microbiological culture and the selection of samples was made by the physicians. We used commercial blood culture bottles (BacT/ALERT, bioMérieux, Marcy l'Etoile, France) to assess bacteremia and sterile cotton (Transswab, MWE, Corsham, England) for superficial infections, urine samples were collected in sterile single-use pots for microbiological culture.

### Microbiological analyses

Standard culture based methods were used for species identification (API Test stripes, bioMérieux, France and BBL Enterotubes or BBL Oxi/Ferm Tube, BD, Germany). Antimicrobial susceptibility testing was performed using the disk-diffusion method and was reported according to Clinical Laboratory Standards Institute (CLSI) guidelines [7]. Inducible clindamycin resistance was not routinely tested in our laboratory and is therefore not reported. We report the non-susceptibility rates, which include both intermediate and resistant isolates. The production of extended-spectrum beta-lactamase was confirmed in all ceftriaxone resistant Enterobacteriaceae using the double-disks method according to the manufacturer’s instruction (Mast discs, Mast diagnostics, Bootle, UK). This test applies discs of three different beta-lactam antibiotics (ceftazidim 30 μg, cefotaxime 30 μg, cefpodoxime 10 μg) with and without clavulanic acid. Methicillin-resistance was confirmed for all cefoxitin-resistant *Staphylococcus aureus* using a PBP2a-agglutination test (PBP2’ Test Kit, Oxoid, Japan). As part of a clinical trial requirement, the microbiology laboratory at the HAS successfully participates in regular external quality assurance (EQA) programs addressing species identification and susceptibility testing. The EQA is part of the WHO/NICD Proficiency Testing Scheme, and is organized by the Contract Laboratory Services (CLS), Johannesburg, South Africa.

### Statistics

In our analysis, children (≤ 18 years old) and adults (> 18 year old) were assessed separately. Types of infection were categorized as "bloodstream infection" (sepsis, bacteremia), "ear, eye, nose, throat infection" (pharyngitis, upper-respiratory tract infection, conjunctivitis, otitis), "surgical site infection", "skin and soft tissue infection" (pyoderma, impetigo, pyomyositis, abscesses, ulcer), "urinary tract infection" and "wound infection" [8]. All data were entered in one excel spreadsheet and analyzed using 'R’, version 2.13.1 (http://www.cran.r-project.org) and the package "epicalc". Categorical variables were compared using Chi-square test. The odds ratio (OR) and the 95% confidence interval (95% CI) were calculated to assess the association. The significance level was p < 0.05.

## Results

### Study population

The proportion of children was higher compared to adults (Table 1). Similarly, the isolates from different groups of infections were not equally distributed among children and adults (Table 2). In total, 34.1% (n = 434) of all cases received a pre-sampling antibiotic treatment. Of these, the majority received ampicillin/amoxicillin (37.6%, n = 163), cloxacillin (16.4%, n = 71), ceftriaxone (15.0%, n = 65), gentamicin (14.1%, n = 61), ciprofloxacin (11.8%, n = 51) or cotrimoxazole (3.0%, n = 13).

**Table 1:** Demographic characteristics of the study population

		**Children**	**Adults**
Patients, n (%)	2009	60 (9.4)	91 (23.0)
	2010	132 (20.8)	79 (20.0)
	2011	235 (37.0)	111 (28.1)
	2012	209 (32.9)	114 (28.9)
	Total	636 (100)	395 (100)
Sex, n (%)	Female	289 (46.5)	263 (70.5)
Mean age in years (range)		3.6 (0–18)	37.8 (18.3-89.7)
No of different species per patient (± SD)		1.1 (0.48)	1.3 (0.68)

**Table 2:** Distribution of isolates from various infections

	**Children, n (%)**	**Adults, n (%)**	**OR (95% CI)**	**p-value**
Bloodstream infection	126 (17.0)	81 (15.3)	1.1 (0.8-1.6)	0.4
Ear, eye, nose, throat infection	167 (22.6)	6 (1.1)	25.5 (11.3-70.9)	<0.0005
Surgical site infection	35 (4.7)	106 (20.0)	0.2 (0.1-0.3)	<0.0005
Skin and soft tissue infection	155 (21.0)	37 (7.0)	3.5 (2.4-5.3)	<0.0005
Urinary tract infection	70 (9.5)	94 (17.7)	0.5 (0.3-0.7)	<0.0005
Wound infection	76 (10.3)	68 (12.8)	0.8 (0.5-1.1)	0.2
Others	111 (15.0)	139 (26.2)	0.5 (0.4-0.7)	<0.0005
Total	740 (100)	531 (100)	-	-

### Bacterial spectrum

In total, 1271 isolates were included in this study comprising isolates from 2009 (n = 234), 2010 (n = 273), 2011 (n = 397) and 2012 (n = 367). Noteworthy, adults and children frequently share the three most common bacterial pathogens but the distribution of these pathogens was different in both groups. For instance, the most frequent isolate from blood cultures in children was *Klebsiella pneumoniae* (16.7%, n = 21) followed by *S. aureus* (11.9%, n = 15) and *Salmonella enterica* (11.1%, n = 14). Similarly, *S. aureus* was most frequently found in blood cultures from adults (18.5%, n = 15) followed by *S. enterica* (14.8%, n = 12) and *Streptococcus pneumoniae* (12.3%, n = 10, Table 3). The spectrum of bacterial pathogens was highly diverse for the majority of infections. For instance, the three most frequent species comprised only one half of all pathogens isolated from blood cultures, ear, eye, nose and throat infection (*S. aureus*, *Pseudomonas aeruginosa*, *Proteus mirabilis*) or surgical site infections (*S. aureus*, *P. mirabilis*, *Escherichia coli* and *Enterococcus faecalis*, respectively, Table 3). The bacterial spectrum was less diverse in skin and soft tissue infections as *S. aureus* dominated in children (74.8%, n = 116) and adults (75.7%, n = 28, Table 2). Urinary tract infections were dominated by *E. coli*, *K. pneumoniae* and *P. mirabilis* both in children and adults. Wound infections in children were frequently caused by *S. aureus* (46.1%, n = 35) and *Streptococcus pyogenes* (26.3%, n = 20, Table 2).

**Table 3:** The three most frequent pathogens from major infections in Gabonese children and adults

**Infection**	**Pathogen**	**Children, n (%)**	**Adults, n (%)**
Bloodstream infection	*Klebsiella pneumoniae*	21 (16.7)	-
	*Staphylococcus aureus*	15 (11.9)	15 (18.5)
	*Salmonella enterica*	14 (11.1)	12 (14.8)
	*Streptococcus pneumoniae*	-	10 (12.4)
	Others	76 (59.5)	44 (54.3)
Ear, eye, nose, throat infection	*Staphylococcus aureus*	37 (22.2)	1 (16.7)
	*Pseudomonas aeruginosa*	15 (9.0)	1 (16.7)
	*Proteus mirabilis*	14 (8.4)	1 (16.7)
	Others	101 (60.5)	3 (50.0)
Surgical site infection	*Staphylococcus aureus*	9 (25.7)	33 (32.0)
	*Escherichia coli*	-	15 (14.2)
	*Enterococcus faecalis*	6 (17.1)	-
	*Proteus mirabilis*	4 (11.4)	7 (6.6)
	Others	16 (45.7)	51 (48.1)
Skin and soft tissue infection	*Staphylococcus aureus*	116 (74.8)	28 (75.7)
	*Streptococcus pyogenes*	31 (20.0)	1 (2.7)
	*Klebsiella pneumoniae*	2 (1.3)	2 (5.4)
	Others	6 (3.9)	6 (16.2)
Urinary tract infection	*Escherichia coli*	28 (40.0)	36 (38.3)
	*Proteus mirabilis*	9 (12.9)	4 (4.3)
	*Klebsiella pneumoniae*	4 (5.7)	19 (20.2)
	Others	29 (41.4)	35 (37.2)
Wound infection	*Staphylococcus aureus*	35 (46.1)	15 (22.1)
	*Streptococcus pyogenes*	20 (26.3)	-
	*Pseudomonas aeruginosa*	7 (9.2)	-
	*Escherichia coli*	-	8 (11.8)
	*Proteus mirabilis*	-	8 (11.8)
	Others	14 (18.4)	37 (54.4)

### Antibiotic non-susceptibility

The overall proportion of methicillin resistant *S. aureus* (MRSA) was 5.8% (n = 19) and fluctuated during the studied period between 3.9% (n = 2, 2009), 10.9% (n = 7, 2010), 5.2% (n = 5, 2011) and 3.3% (n = 4, 2012). The non-susceptibility rates (methicillin, erythromycin and clindamycin) of *S. aureus* from bloodstream infection were lower than in non-invasive isolates (Tables 4 and 5). The non-susceptibility of streptococci against penicillin was 8.6% (non-invasive *S. pyogenes*), 6.7% (non-invasive *S. pneumoniae,* Table 5) and 25% (*S. pneumoniae* from bloodstream infection, Table 4). Noteworthy, the two major Gram-negative bacteria *K. pneumoniae* and *E. coli* were frequently non-susceptible to combinations of aminopenicillins with beta-lactamase inhibitors. The overall non-susceptibility against third generation cephalosporins in *K. pneumoniae* was 50.6% (n = 43), ESBL-production was confirmed in all but one cases (49.4%, n = 42). The proportion of ESBL-producers among all Enterobacteriaceae throughout the observation period was 15.0% (n = 15, 2009), 18.9% (n = 27, 2010), 13.5% (n = 20, 2011) and 13.2% (n = 15, 2012). Despite its wide use in the hospital and other health care facilities in the region, the non-susceptibility to ciprofloxacin was diverse and ranged from 0% (*Salmonella enterica*) to 97.1% (*K. pneumoniae,* Table 4). The non-susceptibility rates of *P. aeruginosa* were very low, non-susceptibility to ciprofloxacin was reported in only one isolate (2.6%, Table 3).

**Table 4:** Non-susceptibility rates of the most prevalent bacterial pathogens from bloodstream infections in Gabon

**Pathogen (n)**	**Antimicrobial non-susceptibility (%)**										
	**PEN/AMP**^**a**^	**COX**	**AMP + BLI**	**CRO**	**GEN**	**CIP**	**CHL**	**CLI**	**ERY**	**SXT**	**TCY**
*S. aureus* (30)	89.7	0	0	0	0	0	0	0	10	7.8	66.7
*S. pyogenes* (3)	0	-	-	0	-	-	ND	0	0	0	-
*S. pneumoniae* (15)	25	-	-	25	-	-	33.3	14.3	13.3	71.4	ND
*K. pneumoniae* (29)	100	-	92.6	74.1	75	37.9	100	-	-	96.3	ND
*P. agglomerans* (11)	90.9	-	81.8	80	45.4	18.2	50	-	-	80	ND
*E. coli* (16)	81.2	-	62.5	0	6.3	12.5	66.7	-	-	81.2	ND
*S. enterica* (26)	3.9	-	3.9	3.9	0	0	11.1	-	-	4	0

Note: Abbrevations are penicillin/ampicillin (PEN/AMP), cefoxitin (COX), ampicillin plus betalactamase-inhibitor (AMP + BLI), ceftriaxone (CRO), gentamicin (GEN), ciprofloxacin (CIP), chloramphenicol (CHL), clindamycin (CLI), erythromycin (ERY), cotrimoxazole (SXT), tetracycline (TCY), not done (ND). Non-susceptible isolates are shown as the percentage of tested isolates. Data from *P. aeruginosa* and *P. mirabilis* were not reported due to small sample size (n = 2).

^a^Shown is the non-susceptibility of Gram-positives to penicillin and the non-susceptibility of Gram-negatives to ampicillin.

**Table 5:** Non-susceptibility rates of the most prevalent non-invasive bacterial pathogens from Gabon

**Pathogen (n)**	**Antimicrobial non-susceptibility (%)**										
	**PEN/AMP**^**a**^	**COX**	**AMP + BLI**	**CRO**	**GEN**	**CIP**	**CHL**	**CLI**	**ERY**	**SXT**	**TCY**
*S. aureus* (312)	94.2	8.33	8.33	8.33	2.3	9.4	0.9	2.5	17.0	8.3	37.5
*S. pyogenes* (69)	8.6	-	-	0	-	-	9.1	7.8	7.9	56.0	-
*S. pneumoniae* (19)	6.7	-	-	ND	-	-	14.3	10.5	11.1	82.4	ND
*E. faecalis* (19)	0	-	-	-	-	81.3	-	-	-	-	-
*K. pneumoniae* (56)	100	-	51.9	38.9	38.2	21.4	55.6	-	-	60	ND
*P. agglomerans* (6)	83.3	-	75.5	ND	0	0	ND	-	-	0	ND
*P. aeruginosa* (39)	-	-	0^b^	0^c^	0^d^	2.6	-	-	-	-	-
*P. mirabilis* (52)	34.6	-	17.3	2.1	13.5	1.9	44.4	-	-	48.9	ND
*E. coli* (114)	80.4	-	60.3	16.4	14.7	24.1	35.5	-	-	88.1	ND
*S. enterica* (9)	22.2	-	ND	0	0	0	0	-	-	12.5	0

Note: Abbrevations are penicillin/ampicillin (PEN/AMP), cefoxitin (COX), ampicillin plus betalactamase-inhibitor (AMP + BLI), ceftriaxone (CRO), gentamicin (GEN), ciprofloxacin (CIP), chloramphenicol (CHL), clindamycin (CLI), erythromycin (ERY), cotrimoxazole (SXT), tetracycline (TCY), not done (ND). Non-susceptible isolates are shown as the percentage of tested isolates.

^a^Shown is the non-susceptibility of Gram-positives to penicillin and the non-susceptibility of Gram-negatives and *E. faecalis* to ampicillin.

^b^Non-susceptibility to piperacillin/tazobactam.

^c^Non-susceptibility to ceftazidim.

^d^Non-susceptibility to amikacin.

## Discussion

In this retrospective study we analyzed the infection epidemiology and the antibiotic resistance patterns of bacterial pathogens isolated in a secondary care hospital in Gabon, Central Africa. Main findings are the predominance of *S. aureus* in major infections (ear, eye, nose and throat infections, surgical site, wound, skin and soft tissue infections) and a high prevalence of ESBL-producing Enterobacteriaceae, in particular *K. pneumoniae*.

The major cause of bloodstream infections in children was *K. pneumoniae* which is in contrast to one report from Ghana and a large meta-analysis from Africa where *Salmonella* spp. was most frequently found [9,10]. An empirical treatment with cotrimoxazole and sometimes even ciprofloxacin before taking blood cultures in our hospital might explain this difference as non-susceptibility rates of *S. enterica* were low against cotrimoxazole (4–12.5%%) and ciprofloxacin (0%) in our setting (Tables 4 and 5).

The predominance of *S. aureus* in bloodstream infections in adults (18.5%, Table 3) is in contrast to a large meta-analysis of community-acquired bloodstream infections as *S. aureus* was only reported in 5.4% of cases [10]. This high proportion in our study is unclear and could be due to the frequent pre-sampling empirical treatment with ampicillin/amoxicillin (37.6% of all patients received ampicillin/amoxicillin), against which *S. aureus* is highly resistant in Gabon (94.2% and 89.7%, respectively, Table 3) [11]. However, one prospective study from Tanzania also reports *S. aureus* as the main pathogen of bloodstream infection in newborns between 7–28 days of age [12].

We found a non-susceptibility to penicillin in *S. pyogenes* of 8.6% (Table 5) which has not yet been reported in sub-Saharan Africa. In general, a universal susceptibility to penicillin is assumed [13]. However, determination of the minimal inhibitory concentration using the microdilution methods was not available to confirm penicillin resistance in *S. pyogenes*.

The high diversity of pathogens isolated from bloodstream infections, ear, eye, nose, throat and surgical site infections challenges an empirical antibiotic therapy. We strongly recommend species identification and susceptibility tests in these cases. Our data can be used to guide empiric therapy for skin and soft tissue as well as wound infections which were predominantly caused by *S. aureus* and *S. pyogenes*. Based on our survey, the susceptibility rate of all isolates from wound infections is 76.7% (clindamycin) and 75.1% (cephalosporin, cloxacillin) in children and 76.5% (clindamycin) and 74.1% (cephalosporin, cloxacillin) in skin and soft tissue infections in adults. Therefore the coverage rate in skin and soft tissue infections in children would be 91.6% (clindamycin) and 90.5% (cephalosporin, cloxacillin). If microbiological diagnostics are not available, we recommend clindamycin or a combination of amoxicillin/clavulanate or cefuroxime or cloxacillin in mild to moderate wound or skin and soft tissue infections [14]. Intravenous antibiotic agents should be considered in severe infections. The prevalence of the *S. aureus* protein toxin Panton-Valentine leukocidin (PVL) is high in Gabon (44.7-55.9%) and can be associated with abscesses [15,16]. We are therefore in favor of clindamycin as it reduces the production in PVL even in sub-inhibitory concentrations *in vitro *[17].

The MRSA rate of 5.8% is lower compared to a meta-analysis of *S. aureus* isolates from Central Africa (27.7%) and another study on clinical *S. aureus* isolates from this region (11.1%) or in a study on neonatal bloodstream infection on Tanzania (28%) [3,12,15]. The high rate of ESBL-producing *K. pneumoniae* (49.4%) is alarming as *K. pneumoniae* was frequently isolated from bloodstream infection, skin and soft tissue infection or urinary tract infection (Table 3). Data on the prevalence of ESBL-producers from clinical samples are scarce in Central Africa. One report from 2005 shows a prevalence of ESBL-producing Enterobacteriaceae of 12% in Cameroon with a predominance of *K. pneumoniae* (18.8%) [18]. Several reports from Tanzania show a higher proportion of ESBL-producing Enterobacteriacea (29.2-50%) [19,20]. However, there is evidence for an increasing prevalence of ESBL-producers, considering carrier rates of 33.6% in children admitted to the HAS in Lambaréné, Gabon [21]. This observation has already direct impact in the choice of drugs.

Our report on bacterial spectra of major infections and antimicrobial non-susceptibility patterns enables a more rational use of antibiotics. This is of outmost importance, not only for patients in Gabon but also for travelers returning from the tropics which have acquired infections in the visited country [22]. Our study provides the basis for future prospective studies for instance on invasive infections and clearly points out the need for a more rational use of antibiotic agents paired with improved education of health care workers on the use of anti-infective drugs as part of antibiotic stewardship programs.

Although the data we present are very valuable in a setting where information on antimicrobial resistance is severely limited, some limitations need to be addressed. First, based on the reports of microbiological findings we were not able to distinguish community from hospital acquired infections. The source of infection is therefore blurred. Second, some samples have also been taken after starting an antibiotic treatment, which may lead to selection and over-representation of resistant isolates. Third, we were unable to provide further epidemiological data (e. g. place of residence, onset of disease). Fourth, the collection of samples for microbiological culture was not systematic but based on the physician’s judgment which might be a sampling bias.

## Conclusion

Our analysis provides important data of bacterial spectra and antimicrobial resistance in a semi-urban setting in Africa. *S. aureus* is the predominant pathogen and the high proportion of ESBL-producing *K. pneumoniae* is alarming. The high diversity of pathogens challenges an empirical use of antibiotics. A countrywide continuous sentinel surveillance is therefore urgently needed.

## Abbreviations

CLS: Contract laboratory services; CLSI: Clinical laboratory standards institute; EARSS: European antimicrobial resistance surveillance system; EQA: External quality assurance; ESBL: Extended-spectrum beta-lactamase; HAS: Albert Schweitzer Hospital; MRSA: Methicillin resistant *Staphylococcus aureus.*

## Competing interests

The authors declare that they have no competing interests.

## Authors’ contributions

AA, LF, HK and FS carried out microbiological studies, AA, KK, MAH, BCK, MPG, GP, SI, PGK and FS were involved in the study conception and design and data analysis. All authors read and approved the final manuscript.

## Pre-publication history

The pre-publication history for this paper can be accessed here:

http://www.biomedcentral.com/1471-2334/13/455/prepub
